# Partial versus radical nephrectomy for T1b-2N0M0 renal tumors: A propensity score matching study based on the SEER database

**DOI:** 10.1371/journal.pone.0193530

**Published:** 2018-02-28

**Authors:** Mengping Zhang, Zhijian Zhao, Xiaolu Duan, Tuo Deng, Chao Cai, Wenqi Wu, Guohua Zeng

**Affiliations:** 1 Department of Oncology, The First Affiliated Hospital of Sun Yat-sen University, Guangzhou, China; 2 Department of Urology, Minimally Invasive Surgery Center, The First Affiliated Hospital of Guangzhou Medical University, Guangzhou, China; Sun Yat-sen University, CHINA

## Abstract

**Purpose:**

Controversy continues on the tailored therapy for patients with larger renal cell carcinoma (RCC). We investigated whether partial nephrectomy (PN) can improve patient prognosis compared to radical nephrectomy (RN) and the indications for each approach in patients with T1b-2N0M0 RCC.

**Materials and methods:**

A total of 9907 patients were identified from the Surveillance, Epidemiology, and End Results database from 2004 to 2012. Propensity scores were used to balance the selection bias of undergoing PN. Overall (OS) and cancer-specific survival (CSS) of patients undergoing PN and RN were compared. Cases were subdivided to investigate the advantages of each procedure.

**Results:**

Overall, 1418 (14.3%) patients underwent PN. Before matching, PN led to better OS and CSS than RN in both Kaplan-Meier analysis and Cox regression (each p<0.01). For 1412 matched cohorts, PN was no longer associated with significantly better OS (HR: 1.19, 95% CI: 0.98–1.44), but still with a better CSS (HR: 1.66, 95% CI: 1.18–2.27) compared with RN. Further subgroup analysis indicated that patients, who were male, single living, old than 65 years, with T1b stage or clear-cell histologic type, may obtained more oncologic benefit from PN compared to RN.

**Conclusions:**

When tumor localization and technical feasibility have been taken into account, similar long-term survival was achieved in overall among two nephrectomy modalities, but patients, who were male, old than 65 years, with T1b stage or clear-cell histologic type, got a better survival after receiving PN compared to RN.

## Introduction

Renal cell carcinoma (RCC) represents 2–3% of all cancers and 85–93% of renal malignant tumors[1]. Nephrectomy remains the most common treatment for clinically localized RCC. In addition, nephron-sparing surgery (NSS) or partial nephrectomy (PN) has been rapidly adopted in recent years and has been an accepted treatment modality in case of patient anephric or at high risk for subsequent renal replacement therapy[2]. PN also has become a viable alternative for treating renal masses < 4 cm in patients with a normal contralateral kidney, with encouraging short-term and long-term oncological outcomes[2,3].

However, the EAU guidelines for management of RCC do not make strong recommendations in favor of PN or radical nephrectomy (RN) for localized RCC > 4 cm[2]. Preservation of renal function without compromising the oncologic outcome should be the most important goal in the decision-making process for RCC treatment. Growing evidence, mainly from retrospective studies, indicates that PN may obtain oncologic outcomes similar or superiority to RN in larger renal masses (T1b–2), and their data were further systemically evaluated in a meta-analysis to compare the renal functional, oncologic, and perioperative outcomes[4]. However, the meta-analysis has many limitations[4]. It is not clear whether these disparate results are related to selection bias in observational studies or inadequate statistical power with small size of patients or short follow-up. These inconsistent results also raise the question of whether and how individual patient and tumor characteristics may impact the efficacy of nephrectomy modalities. Furthermore, whereas meta-analysis of homogeneous studies are the highest form of evidence, poorly conducted meta-analysis create confusion and serve to harm the patient[5].

In this study, we used the population-based Surveillance, Epidemiology, and End Results (SEER) database and the propensity score-matching analysis to compare the oncologic outcomes of patients with T1b-2N0M0 RCC after partial or radical nephrectomy.

## Methods

### Ethics statement

We selected patients from the SEER database released in Nov 2016, which includes cancer registries covering 28% of the U.S. population[6]. The data released by the SEER database do not require informed patient consent since cancer is a reportable disease in the United States, so our study does not require an ethics statement. We obtained the permission to access the SEER database with the ID number 11704-Nov2016 via Internet access method.

### Patients selection

In order to identify eligible patients, we use the inclusion criteria as follows: adult patients ≥ 18 years old, unilateral renal cancer, kidney cancer (ICD-O-3 site code C64.9) as the first and only cancer diagnosis, 2017 TNM classification system T1b-2N0M0, diagnosis from January 2004 and December 2012, treated with partial nephrectomy (Code 30) or radical nephrectomy (Code 40 and 50), and at least 3 months’ survival time after surgery. We use the SEER*stat version 8.3.4 to generate a case-listing file.

Demographic characteristics included age at diagnosis, gender, race, ethnicity, and marital status, patient ID, tumor sequence number. We divided age at diagnosis as a binary variable classified into two groups: 18–65 years and >65 years. Tumor statistics included laterality, diagnostic confirmation, behavior code, histological grade, histologic subtype, pathologic tumor size, regional lymph node (LN) status (2004+), tumor extension (2004+), mets at dx (2004+) status, surgical approach codes, years of treatment, follow-up time. Tumor stage was recoded according to the 2017 TNM classification system[7]. Finally, a total of 9907 T1b-2N0M0 patients with primary RCC as the only one tumor was included in our study. Of these patients, 1418 were treated with PN and 8489 with RN.

### Statistical analysis

Due to potential differences in patients undergoing PN and RN, the propensity score-matching was used to balance the potential probability of being assigned to a treatment group. The logistic regression model included age at diagnosis, sex, tumor size, pathological grade, and histology type, and marital status was used to calculate the propensity scores. Mean propensity-score for PN group was 0.207, and 0.132 for RN group (*p*<0.001). 1: 1 pair matching without replacement was implemented by nearest neighbor matching method with caliber of 0.02. After matching, 1412 pairs of patients were included after applying propensity scores, and there was no significant difference in mean propensity-score between two group (both group of propensity-score = 0.207, *p* = 0.992), and all standardized differences were well below 10%.

Demographic, clinical and pathologic data were compared using independent t-tests for continuous variables and Pearson’s chi-square tests for categorical variables. Overall survival (OS) and cancer-specific survival (CSS) were the primary and secondary outcomes of interest. For OS, patients alive at the end of follow-up (December 31 2014) were censored. For CSS, patients alive or dead due to other than cancer-specific causes were censored. The Kaplan–Meier method was used to compare unadjusted survival of patients undergoing PN or RN with a log-rank test in both full and paired cohorts. Cox proportional hazard models are used to estimate hazard ratio (HR). HR and 95% confidential interval (CI) were reported. Multivariable Cox proportional hazards models included covariates with p value < 0.1 on univariate analyses were used to decide variables that remained in the final model.

Secondary subgroup analyses were performed using univariate Cox proportional hazard model estimated the HRs of PN versus RN and a forest plot was created to better present each prognostic factor’s effect on OS and CSS, which stratified to patients of different gender (female, male), age (≤ 65, > 65 years), T stage (T1b, T2a, T2b), pathological grade (well, moderate, and poor differentiated + undifferentiated), histology subtype (clear-cell, papillary, chromophobe, and others/unknown malignant RCC), race (black or white), and marital status (married or unmarried).

These above statistical analyses and propensity score-matching were performed with SPSS version 23.0 (IBM SPSS Statistics, Chicago, IL, US). Two-sided *p*-value < 0.05 was considered as statistically significant.

## Results

We examined 9907 patients undergoing nephrectomy for T1b-2N0M0 RCC from 2004 to 2012. The baseline characteristics are reported in **Table 1**. Patients undergoing PN were more likely to have smaller tumor size (*P*<0.001), low T stage (*p*<0.001), a significantly lower proportion of clear-cell RCC (*p*<0.0001) and male predominant (*p* = 0.002) than RN patients. After propensity-score matching, preoperative characteristics were well balanced (**Table 1**). However, PN arm was still more likely to have lower proportion of clear-cell RCC (*p*<0.05).

**Table 1:** Patient baseline demographics and pathological characteristics. Full sample and propensity score matched cohorts.

	Full Sample			PS- Matched Cohort		
	PN	RN	Std.	PN	RN	Std.
	n = 1418	n = 8489	Diff	n = 1412	n = 1412	Diff
**Tumor size (mm)**	57.4±29.2	72.0±32.8	-14.7	57.4±29.3	57.2±17.7	0.26
**Mean ± SD age, years**	59.1±12.6	59.9±12.7	-0.8	59.2±12.5	59.0±12.3	0.19
**Age at diagnosis,years**						
** ≤65**	961(67.8)	5647(66.5)	0.90%	955(67.6)	992(70.3)	-1.50%
** >65**	457(32.2)	2842(3.5)	-0.90%	457(32.4)	420(29.7)	1.50%
**Sex,No.(%)**						
** Female**	477(33.6)	3212(37.8)	-3.00%	477(33.8)	431(30.5)	1.90%
** Male**	941(66.4)	5277(62.2)	3.00%	935(66.2)	981(69.5)	-1.90%
**T stage,No.(%)**						
** T1b**	1232(86.9)	5255(61.9)	18.3%	1226(86.8)	1223(86.6)	0.20%
** T2a**	138(9.7)	2165(25.5)	-13.0%	138(9.8)	145(10.3)	-0.40%
** T2b**	48(3.4)	1069(12.6)	-10.1%	48(3.4)	44(3.1)	0.40%
**Grade,No.(%)**						
** I**	145(10.2)	819(9.6)	0.7%	145(10.3)	91(6.4)	3.70%
** II**	652(46)	4024(47.4)	-1.0%	652(46.2)	713(50.5)	-2.30%
** III+UD**	449(31.7)	2800(33)	-1.0%	446(31.6)	466(33)	-0.80%
** Unknown**	172(12.1)	846(10)	2.5%	169(12)	42(10.1)	1.60%
**Histology type,No.(%)**						
** Clear-cell**	725(51.2)	5169(60.9)	-6.9%	725(51.3)	856(60.6)	-5.00%
** Papillary**	298(21.0)	914(10.8)	10.9%	298(21.1)	144(10.2)	8.00%
** Chromophobe**	131(9.2)	757(8.9)	0.4%	130(9.2)	125(8.9)	0.30%
** Others +Unknown**	264(18.6)	649(19.4)	-0.7%	259(8.3)	287(20.3)	-1.30%
**Laterality,No.(%)**						
** Left**	684(48.2)	4223(49.7)	-1.1%	680(48.2)	695(49.2)	-0.60%
** Right**	734(51.8)	4266(50.3)	1.1%	732(51.9)	717(50.8)	0.60%
**Years at diagnosis,No.(%)**						
** 2004–2008**	517(36.5)	4828(56.9)	-14.3%	517(36.6)	492(34.8)	1.00%
** 2009–2012**	901(63.5)	3661(43.1)	14.3%	895(63.4)	920(65.2)	-1.00%
**Race,No.(%)**						
** Black**	205(14.5)	938(11)	3.7%	204(14.4)	159(11.3)	2.50%
** White**	1075(75.8)	6716(79.1)	-2.8%	1071(75.8)	1113(78.8)	-1.90%
** Others+Unknown**	38(9.7)	835(9.8)	-0.1%	137(9.7)	140(9.9)	-0.20%
**Hispanic,No.(%)**						
** yes**	191(13.5)	1306(15.4)	-1.9%	191(13.5)	215(15.2)	-1.30%
** no**	1227(86.5)	7183(84.6)	1.9%	1221(86.5)	1197(84.8)	1.30%
**Marital status,No.(%)**						
** Married**	873(61.6)	5187(61.1)	0.3%	869(61.5)	875(62)	-0.20%
** Unmarried**^**a**^	491(34.6)	2887(34)	0.5%	489(34.6)	462(32.7)	1.10%
** Unknown**	54(3.8)	415(4.9)	1.8%	54(3.8)	75(5.3)	1.90%
**Median follow-up(IQR)**	51(35–79)	65(40–93)	-	51(35–79)	53(35–77)	-

NOS = no other specific, UD = undifferentiated

^a^Unmarried = Including divorced, separated, single (never married), unmarried, domestic partner and widowed.

**Fig 1:**
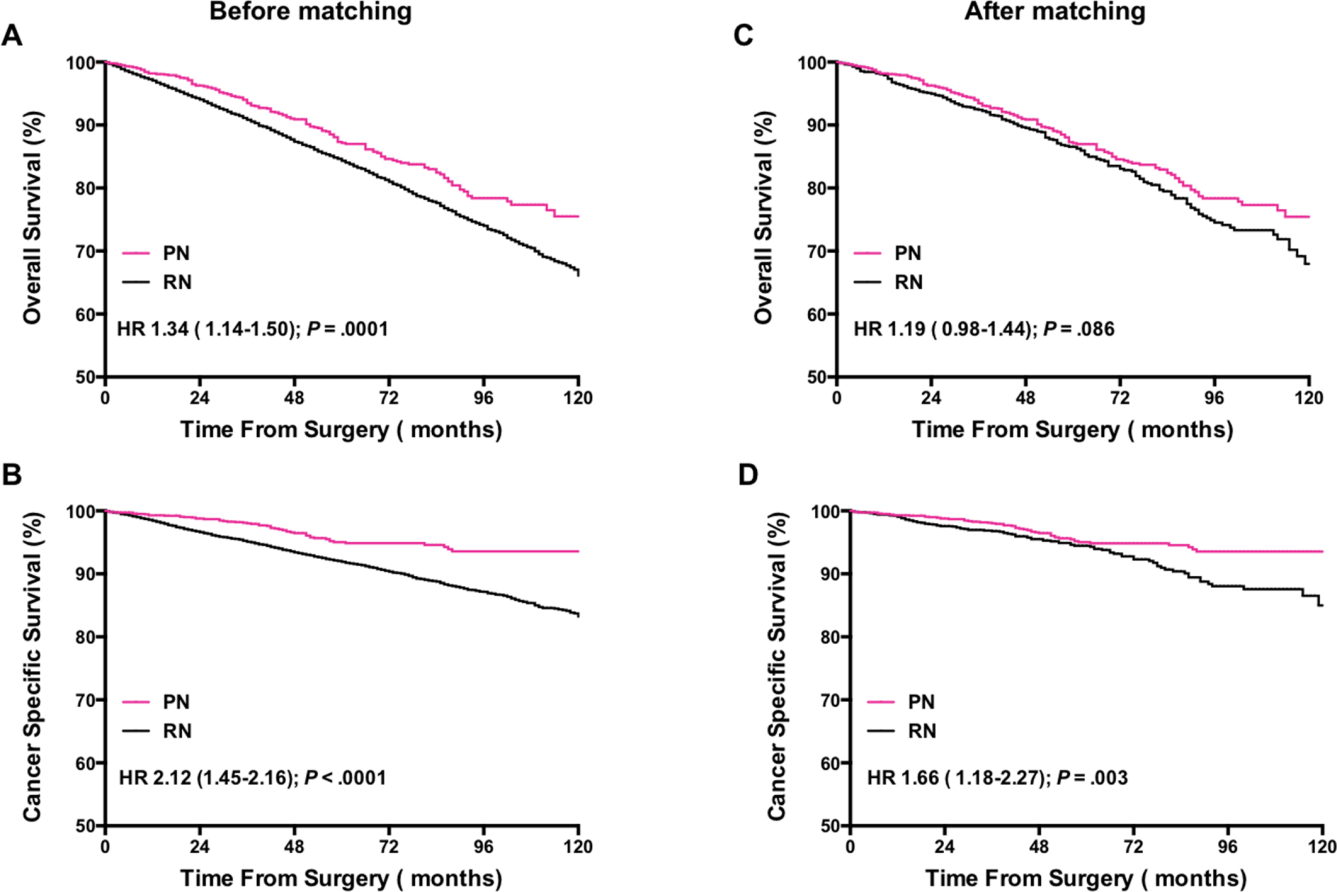
Kaplan-Meier survival curves demonstrating patients’ overall survivals(OS), and cancer-specific survivals (CSS) according to patients’ nephrectomy modalities (partial versus radical) before and after propensity matching.

Before matching, the median (IQR) follow-up interval was 51 (35–79) months and 65(40–93) months for PN and RN, respectively. The median (IQR) follow-up of death cases was 42 (21–69) months in the overall cohort. As the plots illustrated (**Fig 1A and 1B**), OS and CSS were both worse in RN patients (HR: 1.34, 95% CI: 1.14–1.50, *p*<0.001 and HR: 2.12, 95% CI:1.45–2.16, *p*<0.001 respectively) than in PN patients.

In order to further investigate the effects of PN vs. RN in OS and CSS, a multivariate analysis by Cox proportional hazards model was performed. On multivariate analysis, PN was an independent favor predictor of OS and CSS (HR = 1.30, 95% CI: 1.12–1.52, and HR:1.68, 95% CI:1.29–2.21), each p < 0.001). In addition, the multivariate analysis validated that age at diagnosis, sex, grade, T stage, and histology subtype were associated with outcomes (**Table 2**).

**Table 2:** Multivariate analysis of overall survival (OS) and cancer-specific survival (CSS) predictors using cox proportional hazard model.

	OS		CSS	
	HR (95% CI)	p-Value	HR (95% CI)	p-Value
**Nephrectomy type, No.(%)**				
**PN**	1		1	
**RN**	1.30(1.12–1.52)	0.001	1.68(1.29–2.21)	<0.0001
**Age at diagnosis (years),No.(%)**				
**≤65**	1		1	
**>65**	2.75(2.51–2.75)	<0.0001	2.08(1.82–2.38)	<0.0001
**Sex, No.(%)**				
**Female**	1		1	
**Male**	1.28 (1.16–1.40)	<0.0001	1.17(1.02–1.35)	0.028
**T stage, No.(%)**				
**T1b**	1		1	
**T2a**	1.29(1.12–1.50)	0.001	1.60(1.33–1.92	<0.0001
**T2b**	1.55(1.36–1.77)	<0.0001	2.85(2.39–3.38)	<0.0001
**Grade, No.(%)**				
**I**	1		1	
**II**	1.08(0.88–1.34)	0.366	1.20(0.85–1.70)	0.292
**III+UD**	1.21(1.03–1.43)	0.016	1.26(0.97–1.64)	0.076
**Histology type, No.(%)**				
** Clear-cell**	1		1	
** Papillary**	0.89(0.81–1.00)	0.055	0.85(0.732–1.0)	0.052
** Chromophobe**	0.49(0.39–0.62)	<0.0001	0.33(0.23–0.48)	<0.0001
** Others +Unknown**	0.99(0.85–1.16)	0.949	0.76(0.60–0.96)	0.021

Within the propensity-score matched cohort, PN was associated with no significant different outcomes in OS (HR: 1.19, 95% CI: 0.98–1.44, p = 0.086), but still with a better CSS (HR: 1.66, 95% CI: 1.186–2.27, p = 0.003) compared with RN (**Fig 1C and 1D**).

**Fig 2:**
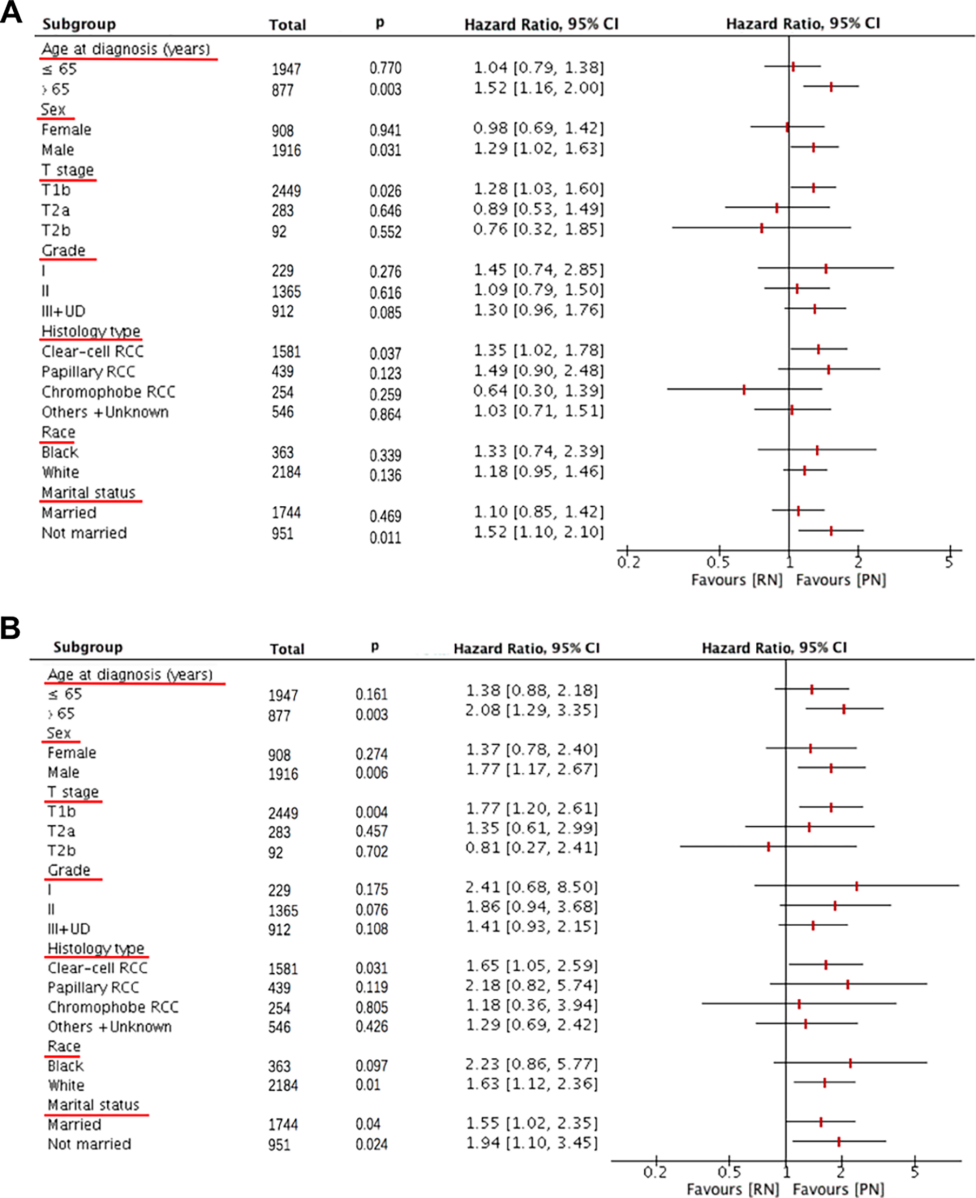
Forest plot of hazard ratios (HRs) for partial (PN) versus radical (RN) nephrectomy in the subgroup analysis. The diamond on the X-axis indicates the HR and the 95% confident interval (CI) of each subgroup.

On secondary analysis, a forest plot of HRs was used to illustrate the exploratory subgroup analyses when stratifying patients by age, sex, T stage (size), pathological grade, histology type, marital status, and race. For both OS and CSS, consistent negative results were found in most subgroups, however, prognosis for patients with older age at diagnosis >65 years, male, T1b stage, clear-cell RCC subtype, and unmarried status were worse in patients undergoing radical nephrectomy than in patients undergoing partial nephrectomy (**Fig 2A**). Moreover, patients undergoing radical nephrectomy were presented with worse CSS in patients with white race (**Fig 2B**).

## Discussion

We report the largest series analysis (about 1400 cases for each group) comparing PN to RN for larger (T1b and T2) renal masses, which can contribute to the ongoing debate about the role of nephron-sparing surgery and provide key information for contemporary evidence-based surgical decision making. In this study, our findings showed that PN has not different overall survival than RN for these T1b-2N0M0 patients. However, we were able to identify some subgroups of population that obtained an oncologic benefit from PN, among patients who were single living, old than 65 years, and male, or with T1b stage and when systematically examining increasing thresholds of risk for clear-cell histologic type. In addition, in patients with pathologic T1-2 RCC, cancer-specific survival after PN occurred more commonly compared to after RN. However, as a low occurrence of cancer-specific death in these patients occurred post-surgery even 5 years, a longer follow-up time is required.

While earlier studies have established the feasibility of PN for smaller (T1a) tumors, more recent studies suggest PN for T1b tumors can be safe, with similar oncologic outcomes and no increased risk of complications[8]. This has led to PN being increasingly performed for larger tumors[9]. A few studies have shown that open and laparoscopic PN for T2a tumors is safe with low perioperative complication rates[10]. Still however, RN is utilized for many cT1b tumors and is the recommended treatment for T2a (7 to <10 cm) and greater tumors[8,11,12].

Tailored therapy for patients with larger (T1b and T2) renal masses s is still an issue of debate and existing relevant reports offered inconsistent findings[4]. In a retrospective study on 873 and 286 patients undergoing PN and RN for T1b RCC, there was not a statistically significant association between type of surgery and OS (p = 0.7), but nearly twice risk of CSS (HR1.97) after adjusting for age, renal function and tumor factors[13]. Weight et al identified PN as an independent favor predictor of OS (HR 0.30) among a cohort composed of 212 PN and 298 RN patients with T1b non-metastatic RCC[14]. However, others have noted no significant impact on OS and CSS of RCC patients between PN and RN approach [4]. Unfortunately, most of their studies were focused on the T1b tumors. From now on, only three studies compared PN to RN in the specific case of T2 (>7 cm) tumors[9,12,15]. Two separated single institutional studies demonstrated that PN for T2 or greater renal cell carcinoma preserves renal function and did not have any significant influence on OS and CSS[9,12]. But a multi-institutional study of 925 patients who underwent nephrectomy treatment for >7cm RCC revealed that PN was an independent poor predictor of OS (HR5.3)[15]. Given the confusing role of PN in the management of larger renal tumors (T1b and T2), a meta-analysis including 21 case-control studies (RN 8620; PN 2584 patients) were conducted, which showed that PN was associated with better postoperative renal function, a lower likelihood of tumor recurrence (HR0.6), cancer-specific mortality (HR0.58), and all-cause mortality (HR0.67)[4]. However, the retrospective nature caused the different preoperative tumor parameters, small size of PN patients or with a relative short follow-up time were their major limitations of the meta-analysis. To our best knowledge, this present study contained the largest number of patients compared to other published studies.

Present study showed that OS was not influenced by PN or RN in the matched cohort (HR1.19, p = 0.086), which was not coincide to the univariate analyses and multivariable regression models before matching. The potential explanations can be offered on the superiority of the propensity score matching for reducing the effects of confounding compared to regression adjustment methods in observational studies[16]. Among several practical reasons, the most important one is the distribution of observed baseline covariates will be the same in propensity score analysis while it is much more difficult to determine they have been correctly specified in multivariable regression model[16]. Second, insufficient outcomes may be observed in the regression model when outcomes (when binary or time-to-event in nature) are rare[17].

Besides tumor size, the other factors may also be important factors that affects the outcome of partial nephrectomy. For example, Kopp et al reported a study specifically looking at comparative outcomes of PN and RN for clinical T2 renal masses after adjusting for tumor complexity as based on the RENAL score >10 or not[18]. So in our study, whether the real therapeutic effect of PN was underestimated or overestimated by including elderly patients [8], or including patients in stage of T2a or T2b [12], or patients having different histology subtype [19,20], remains unclear. Thus, we performed a subgroup analysis using the matched cohort.

Histology subtype of RCC has recently been proven to be a crucial factor that affects the survival of patients who undergo surgery treatment, hence we integrated this parameter as well as other prognostic confounders into the subgroup models for better adjustment. One pilot study found that in patients with pathologic T1a RCC, recurrence after PN occurred more commonly in papillary RCC compared with clear-cell RCC[19]. Another study found that the disease-free survival curve between clear cell and non-clear cell of the most common subtype was statistically significant in patients with tumor size >4 cm[20]. Based on the results of our study, the clear-cell RCC patients would potentially receive more therapeutic benefit of PN than RN. Fortunately, with the advance development of imaging technology, current clinical imaging methods are developed in the detection and correct characterization of different RCC subtypes, such as using the Grating-based X-ray Phase-contrast CT[21], multiphasic multidetector computed tomography (MDCT) scan[22] and 18F-fluorodeoxyglucose-PETCT[23].This clinical imaging information may contribute to treatment plan by the urologist.

One study reported that OS and CSS were equivalent between PN and RN for T1-T2 renal masses in the elderly patients[8]. However, PN group has smaller tumors (tumor diameter 2.8 cm vs 5.0 cm, P <0 .001), and the median follow-up was 36 months[8]. Based on the different results compared to our study, we postulate that the discrepancy could be attributed to the different tumor size and short follow-up time, which would potentially override the therapeutic benefit of PN, because over half of the death cases in patients with RCC occurred ≥ 3 years post-surgery, a longer follow-up time is required.

This study had certain limitations. First, the analyses are retrospective in nature; this comes with an unavoidable selection bias that is prevalent in all non-prospective, nonrandomized studies. Second, preoperative renal function is not available in the SEER registry, which make it difficult to balance the higher likelihood of choosing patients with poorer kidney function to receive partial resection. As we know, a poor renal function is associated with higher risk of severe cardiovascular disease and all-cause mortality. Therefore, it may underestimate the role of PN if a higher proportion of preoperative poor renal function of patients in PN group. Third, the lacking of exact tumor location made it hard to estimate whether the lesion was amenable to partial nephrectomy or not [24]. Besides, we were unable to evaluate whether partial nephrectomy would lead to better renal function preservation, a greater chance of receiving systemic therapy, a higher rate of insufficient margins or cancer recurrence. Furthermore, the lack of clinical staging forced us to select patients solely on pathological stage, which would inevitably exclude some clinical stage T1b or T2 cases that found to be pathological stage T3 or distant metastasis. As excluding such patients would falsely improve the survival, the ability of applying the current findings to all clinical stage T1b and T2 patients could be limited. Finally, most notably the lack of information about the co-morbidities, performance status, in the absence of these data points, the selection bias in choosing patients for surgical procedures is not known. It is possible that patients undergoing surgical resection may be relatively healthy at baseline and deemed fit to tolerate operation, however, potential selection bias could not be totally excluded. Despite these limitations, information on important patient-related and tumor-related factors that play a vital role in the decision making was available and analyzed in this study.

In conclusion, when tumor localization and technical feasibility have been taken into account, similar and even better long-term survival can be achieved with PN in patients with T1b-2N0M0 patients compared to RN, especially when patients were male, old than 65 years, with T1b stage or clear-cell histologic type. These results should be further confirmed via prospective randomized trials.
